# Estimating everyday risk: Subjective judgments are related to objective risk, mapping of numerical magnitudes and previous experience

**DOI:** 10.1371/journal.pone.0207356

**Published:** 2018-12-05

**Authors:** Hannah A. D. Keage, Tobias Loetscher

**Affiliations:** Cognitive Ageing and Impairment Neurosciences, School of Psychology, Social Work and Social Policy, University of South Australia, Adelaide, Australia; University of Social Sciences and Humanities, Faculty in Wroclaw, POLAND

## Abstract

We aimed to investigate individual differences that associate with peoples’ acute risk perception for activities such as walking and giving birth, including objective risk and the mapping of numerical magnitudes. The Amazon Mechanical Turk platform was used, with 284 participants recruited (40% female) ranging between 19 and 68 years. Participants had to indicate the positions of (1) the relative death risk of activities on a horizontal-line with ‘very low risk of death’ and ‘very high risk of death’ as left and right anchors respectively and (2), numerical magnitudes on a horizontal-line ranging 0–1000. The MicroMort framework was used to index acute risk of death (one/million chance of dying from an accident). Previous experience with the activities, handedness, along with risk propensity and unrealistic optimism were also measured. Linear mixed-effects modelling was used to investigate predictors of subjective MicroMort judgments. Individuals subjectively judged activities to be riskier if the activity was objectively riskier, if they over-estimated on the numerical task (more so for low-risk activities as compared to high-risk), or if they had not experienced the activity previously. The observed relationship between the number line task and everyday risk judgments is in keeping with the idea of a common magnitude representation system. In conclusion, individuals are able to discriminate between activities varying in risk in an absolute sense, however intuition for judging the relative differences in risk is poor. The relationship between the misjudging of both risks and numerical magnitudes warrants further investigation, as may inform the development of risk communication strategies.

## Introduction

Accurately judging risk is critical to our survival. Errors in judgment are a major cause of deaths worldwide [1] and the social, financial and economic impacts of poor risk judgment are enormous. Risk perception research has focused on judgments, and behaviours (taking/avoiding risks) related to risks in health (e.g., probability of death or ill health [2, 3–5]), finance [6], and gambling [7].

These lines of research have uncovered important insights in how we think about risk. First, we overweight probabilities in decisions relating to small risks and underweight those in decisions relating to medium and large risks [8, 9]. As a result, an increase in a risk of death by 1% may be perceived as big if the baseline risk was 1% (double the risk), but the same 1% risk increase may be perceived as rather trivial if the baseline risk of dying was 55% [8]. These non-linear probability weightings may reflect difficulties reconciling absolute versus relative risks [10–12]. The characteristic shape of probability weights for risk-related decisions has been captured in decision making theories, such as the influential cumulative prospect theory [9].

Second, while decision-making theories such as the prospect theory capture important aspects of risk perception, other factors such as emotion, values, social pressures, and the environment can influence how risks are perceived [4, 13]. Several theories have attempted to integrate roles of non-cognitive factors to predict behaviour under risk and uncertainty: emotion and body-state representation (the somatic marker hypothesis [14, 15]); affect experienced in the moment of a decision (risk as feelings hypothesis [16]), affect specific to the positive and negative qualities of stimuli (affect heuristic [17]), and anticipated regret (negative outcome/emotion) of a poor outcome (regret theory [18]). There are empirical data to support the contribution of affect on risk perception. For example, people with positive affect towards cigarettes perceive the risks associated with cigarettes as lower compared to people with negative affect, and are more likely to be a current cigarette user [19]. Differences in perceptions as a function of a person’s affect illustrates that the understanding of risk varies widely across individuals. A number of other individual differences that predict risk perception, are demographic factors including age [20] and gender [21], along with unrealistic optimism [22], risk propensity [23], and numeracy [24–26].

Given the extensive research on health and financial/gambling risk over the last decades, it may be a surprise that we know little about the accuracy of peoples’ acute risk perception judgments for everyday activities. For example, how accurately can we judge the risk of death for activities such as walking, sky diving, climbing and giving birth? By acute, we mean that the outcome (i.e. death) will be apparent during the activity. If we decide to sky-dive, there is an acute risk of death during the sky-dive, which will be redundant after completion. In contrast, there are activities that carry chronic risks of death (i.e. relevant after completion of the activity). Smoking is associated with increased mortality, and can be conceptualised as a chronic risky behaviour that can be indexed in terms of life-span lost.

A difficulty for investigating the accuracy of everyday risks perception has been the lack of an obvious objective framework on which to compare subjective responses. This difficulty stands in contrast to other fields of risk research. For example, risk perception in health contexts, uses the probability of death or ill health (e.g. as compiled by disease data registries) as the objective comparator [2, 3, 27]; and in financial fields, losses and gains in gambling tasks can be used as an objective comparator [28, 29]. In the current study the concept of MicroMorts is introduced as an objective risk framework to investigate the accuracy of everyday risk perception.

We have around a one in a million chance of dying from an accident or incident every day, and this acute risk is quantified as one MicroMort [30, 31]. That is, MicroMorts are units that index acute risk (i.e. sudden death): one MicroMort is a one-in-a-million chance of death. We increase our risk through our choices of activities, for example, skydiving has a MicroMort value of 10, walking 27 miles has a MicroMort value of one, and giving birth has a MicroMort value of 120 (i.e. 10, 1 or 120 chance in a million chance of dying respectively) [31]. MicroMorts enable us to compare the acute risk of death from various activities, for example, a general anaesthetic and a sky-dive both carry the same acute risk of death, 10 MicroMorts (10 in one million people will die as a result of doing either). This MicroMort framework is being increasingly being used to index health risks and provide a framework for risk communication, including patient consent [31–33]

The primary aim of this study was to investigate the accuracy of everyday risk perception (i.e. the relationship between subjective and objective assessments) using the MicroMort framework. The secondary aim was to identify individual differences underlying this accuracy. A good understanding of how people judge these risks is needed to better communicate objective risks [34–36], with the aim of reducing accidental deaths along with premature disability and ill health. The investigated factors which may associate with an individual’s accuracy were drawn from previously identified factors associated with risk-taking and judgments; and included previous experience with the activity, age, gender, handedness, optimism bias, risk propensity, and aspects of numerical ability [16, 17, 37, 38]. We expected that subjective responses would positively relate to objective risk (MicroMorts) for recreational activities, based on work in financial and health fields. Further, we predicted that lower subjective risk judgments would relate to having undertaken the activities previously, younger age, male gender, high unrealistic optimism and higher risk propensity. Under exploratory investigation were the effects on risk perception of handedness and degree of error on a numerical estimation task. The exploration of handedness was based on suggestions that non-right handers have an increased accident-related injury risk (e.g. [39, 40]). To investigate whether the weighting of low and higher risks activity is related to errors in mapping low and high numbers on space was driven by suggestions that all quantifiable domains are mapped onto a generalized magnitude system in the parietal lobes of the brain [41–43], and that the representation of bigger stimuli in one domain should correlate with representations of bigger stimuli in another domains [43].

## Material and methods

### Participants

Three hundred and two individuals participated on the Amazon Mechanical Turk platform on 31 July 2015. We excluded 18 individuals due to lack of variation on the line judgment tasks (see Statistical Approach section below), meaning there were 284 in the final analyses; n = 115 female (40%) and n = 169 male (60%), age range 19–68, mean age = 34.02 years (95%CI 32.74–35.31). Ninety-one percent were right-handed, the remaining nine percent were ambidextrous or left-handed [44].

### Materials

#### MicroMort acute risk perception task

This task required participants to estimate the acute risk of death for 20 activities (see Table 1), presented one at a time in random order. Participants were required to select a position on a horizontal line (via mouse or touch pad) that they felt corresponded to the relative risk of death of the activity shown; with the anchors “very low risk of death” on the left side and “very high risk of death” on the right side. Each of the 20 responses was recorded and used in analyses, ranging from 0 to 800 (relating to the 800-long pixel line). These data allowed us to investigate if subjective judgments on this line (with the anchors very low risk of death and very high risk of death) were related to objective risk and numerical magnitude mappings.

**Table 1:** Everyday activities judged by participants and their objective acute risk in MicroMorts (risk of number of deaths per 1 million).

Activities	MicroMort (acute risk of death)
Walking 27 miles	1
Cycling 28 miles	1
Riding a motorbike 7 miles	1
Driving 333 miles	1
Train 7,500 miles	1
Commercial aircraft 7,500 miles	1
Light aircraft 15 miles	1
Rock climbing	3
Scuba diving	5
Working any occupation for one year	6
Running marathon	7
Hang Gliding	8
Skydiving	10
Anaesthesia	10
Giving Birth	120
Caesarean Section Birth	170
Coal mining	430
Base Jumping	430
Commercial Fishing	1,020
Climb Mt. Everest	12,000

#### Number line task

This task assessed accuracy of symbolic-number mapping and was adapted from Schley and Peters [45] and Siegler and Opfer [46]. We used the same 11 numbers and the same anchor points at either ends of a horizontal line as Schley and Peters [45]. These eleven numbers were presented to participants (0.1, 0.8, 1.5, 9.5, 23.2, 89.3, 268, 442, 682, 834, and 925), one at a time and in a random order, below which a line was presented with 0 at one end (left) and 1000 at the other end (right). Participants had to indicate the number’s position on the line using their mouse or touch pad (i.e. a virtual mouse on a laptop), dependent on their device.

This numerical line task has been shown to reflect aspects of numeracy. In their study, Schley and Peters [45] reported that better numeracy (as measured by scale developed by Weller et al. [47]) predicted more accurate performance on the number line task: *b* = 0.22, t(74) = 5.87, *p* < .001. Further, they reported that when performance on the number line task was entered into a model predicting diminishing marginal utility (the primary outcome of the study), performance on the numeracy task [47] went from significant to non-significant; again indicating that performance on the two tasks (numeracy and number line) share substantial common variance, and demonstrating the construct validity of the number line task.

We calculated the deviation of each response from the actual number presented (we transformed values of 0–1000 into their 0–800 equivalents, to match the pixel line length), and averaged these across all 11 trials. This created one variable representing the mean error across all trials, with negative values indicating under-estimation and positive values indicating over-estimation. Participants on average performed well on the task, with a mean error of -3.98 (SD = 40.72; relative to the 800-pixel length line), indicative of a slight group-level under-estimation.

#### Previous experience questionnaire

Participants were asked whether they had undertaken the activities included in the MicroMort task, with possible responses being yes, no, or not applicable.

#### Risk propensity scale (RPS)

The RPS is a short general measure of risk-taking tendencies [48]. Participants were asked to indicate the extents to which they agreed or disagreed with seven risk-related statements (e.g. “I do not take risks” and “I take risks regularly”) from one “totally disagree” to seven “totally agree”, in one-unit intervals, which were presented together on one screen. Items 1, 2, 3 and 5 were reverse coded. All responses were totalled, with higher scores indicate greater risk propensity.

#### Unrealistic optimism task

This task was adapted from McKay et al. [49] and required participants to indicate their perceived risk of contracting 16 conditions (acne, flu, eczema, angina, cough, cholera, infarct, ebola, tumour, leprosy, embolism, fever, AIDS, tooth decay, plague, measles) relative to others of the same age, sex and nationality. Responses were: -3 very much smaller, -2 much smaller, -1 somewhat smaller, 0 equal, 1 somewhat larger, 2 much larger, 3 very much larger. Scores were summed together and multiplied by -1; with higher scores representing higher optimism bias.

#### FLANDERS

The FLANDERS measures handedness [44]. Participants were presented with all ten tasks on a screen (writing, eating, brushing teeth, lighting a match, erasing a pencil mark, sewing, buttering bread, hammering, peeling an apple, and drawing) and indicated whether they used the left, right or either hand to complete the task. Scores were summed, with ≤ -5 representing left-handedness, ≥ 5 representing right-handedness, and scores in between representing ambidextrous individuals. We included handedness as a dichotomous variable: right-handed versus left handed/ambidextrous.

### Procedure

This research project has been approved by the Flinders University Social and Behavioural Research Ethics Committee (Project Number 6435). All research was performed in accordance with relevant guidelines, and informed consent was obtained from all participants.

The experiment was listed on the AMT marketplace on 31 July 2015. Participants accepting the task were directed to an external webpage where they were first gave informed consent and then completed the experiment. Before being able to start this task, participants were required to correctly answer a multiple choice question, to ensure they understood the task. They were asked what they were meant to indicate the relative risk of, with response options of: disgust, death (correct), disease, diabetes. Ninety-five percent of participants correctly responded on their first attempt. Moreover, participants could not move forward within the study materials on the webpage if they did not provide an answer to all questions on the current screen. The order of all tests were counterbalanced across participants. The average completion time was 9:41 minutes and participant reimbursement was USD$1.3.

The Mechanical Turk platform has shown to be valid and highly informative method of sourcing participants for psychological studies, enabling data from large and relatively diverse samples to be collected [45, 50, 51]. Cognitive and perceptual data obtained from the Mechanical Turk platform have shown to be as reliable as those collected in the laboratory [52, 53].

### Statistical approach

The STATA 15.1 IC package was used. We excluded 18 participants (5.9% of sample) based on low standard deviations on the line judgment tasks (Numerical Line and MicroMort), indicating they were selecting a very similar line position for each response. Specifically, we standardised the aggregate of standard deviation of responses on both line tasks (separately) and excluded individuals if they scored greater than -3 (i.e. three standard deviations away from the mean); notably, there were no individuals who scored +3 from the mean. We also excluded individual responses—relative to the individual 20 activities—on the MicroMort task if they were an outlier, using a standard definition of 1.5 times the interquartile range below and above quartile 1 and 3 respectively; this resulted in 119 missing values (across total of 5680 subjective judgments, or 2%).

We used mixed effects modelling to investigate predictors of MicroMort subjective judgments (20 in total; ranging from 0 to 800 relating to the pixel length of the response line), which were log-transformed (1 was added to all values prior to log transform, as there were 19 responses of 0), with maximum likelihood estimation. We set ID as a random intercept, which enabled us to model a different “baseline” subjective risk rating for each individual. Given the unbalanced design, with 7/20 activities with MicroMort values of 1, we re-ran analyses including random combinations of only two of these seven in analyses; effects did not change and so we have reported the full model with all activities included.

Notably, we log transformed objective MicroMorts due to their distribution (see Table 1), which when not transformed lead to strongly skewed model residuals. We also log transformed subjective judgments in order to model a linear relationship with objective MicroMorts. Collinearity was assessed with VIF, with all values under 2. Cohen’s *f*2 was used as a measure of effect size, with .020 signalling a small effect size and .150 a medium effect size.

The model equation used in the main analysis was:

$$xtmixed\;subjective_{perception}\;MicroMort\;numerical_{ability}\;MicroMort*numerical_{ability}$$

$$i.previous_{experience}\;age\;gender\;risk_{propensity}\;handedness\;optimism\;\|id:,cov(uns)$$

Subjective perception had 20 repeated measures (for each activity), each representing the log of the location on the 800 pixel line (+1). MicroMorts had 20 repeated measures (for each activity), each being the objective MicroMort value for each activity. Numerical ability was the average error on the number line task. We included an interaction between numerical cognition and MicroMort values. Previous experience was a three level categorical variable: yes, no, not applicable. Age, risk propensity (RPS) and optimism (Unrealistic Optimism Task) were continuous variables, gender (male/female) and handedness (right/left or ambidextrous) were a two level categorical variables. These data have been made available in Supporting Information (S1 Data; data underlying this study).

## Results

### Prevalence of previous experiences

The proportions of participants who had previous experience with activities in the MicroMort task are presented in Table 2. These data provided us with validity check-points, with highly prevalent activities such as walking 27 miles being reported by 98% of participants; and gendered activities being reported so, for example, 44% of females, as opposed to 1% of males, reported giving birth (we decided not to remove males reporting this, as we did not give transgender or other gender options in this survey). Further, uncommon activities such as coal mining were found to have been experienced by only 2% of participants.

**Table 2:** Responses to the previous experiences questionnaire (F = female; M = male; T = total) from n = 284 in analyses.

		Yes		No		Not applicable	
		n	%	n	%	n	%
Walking 27 miles	F	114	99	1	1	0	0
	M	164	97	4	2	1	1
	T	278	98	5	2	1	0
Cycling 28 miles	F	79	69	33	29	3	3
	M	144	85	23	14	2	1
	T	223	79	56	20	5	2
Riding a motorbike 7 miles	F	27	23	85	74	3	3
	M	52	31	110	65	7	4
	T	79	28	195	69	10	4
Driving 333 miles	F	102	89	12	10	1	1
	M	161	95	7	4	1	1
	T	263	93	19	7	2	1
Train 7,500 miles	F	94	82	21	18	0	0
	M	135	80	29	17	5	3
	T	229	81	50	18	5	2
Commercial aircraft 7,500 miles (fly to London)	F	18	16	95	83	2	2
	M	29	17	133	79	7	4
	T	47	17	228	80	9	3
Light aircraft 15 miles (joy flight)	F	12	10	99	86	4	3
	M	17	10	144	85	8	5
	T	29	10	243	86	12	4
Rock climbing	F	17	15	95	83	3	3
	M	48	28	115	67	5	3
	T	65	23	211	74	8	3
Scuba diving	F	6	5	106	92	3	3
	M	20	12	151	83	8	5
	T	26	9	247	87	11	4
Working one year	F	109	95	6	5	0	0
	M	154	91	13	8	2	1
	T	263	93	19	7	2	1
Running marathon	F	10	9	101	88	4	3
	M	11	7	151	89	7	4
	T	21	7	252	89	11	4
Hang Gliding	F	1	1	111	97	3	3
	M	5	3	157	93	7	4
	T	6	2	268	94	10	4
Skydiving	F	2	2	111	97	2	2
	M	7	4	155	92	7	4
	T	9	3	266	94	9	3
Anaesthesia	F	71	62	40	35	4	3
	M	96	57	66	39	7	4
	T	167	59	106	37	11	4
Giving Birth	F	51	44	59	51	5	4
	M	2	1	79	45	91	56
	T	53	19	135	48	96	95
Caesarean Section Birth	F	18	16	85	74	12	10
	M	2	1	79	47	88	52
	T	20	7	164	58	100	35
Coal mining	F	0	0	109	95	6	5
	M	2	1	159	94	8	4
	T	2	1	268	94	14	5
Base Jumping	F	0	0	111	97	4	3
	M	6	4	157	93	6	4
	T	6	2	268	94	10	4
Commercial Fishing	F	8	7	102	89	5	4
	M	33	20	130	77	6	4
	T	41	14	232	82	11	4
Climb Mt. Everest	F	0	0	109	95	6	5
	M	2	1	157	93	10	6
	T	2	1	266	94	16	6

### Correlations between model predictor variables

We investigated the bivariate correlations between the predictor variables gender, age, optimism bias, risk propensity and numerical ability. See Table 3. Optimism bias increased with age (*r* = .137, *p* = .021); and higher risk propensity was associated with being male (*r* = .275, *p* < .001) and being left handed/ambidextrous (*r* = -.205, *p* = .001). Notably, age and gender did significantly correlate (*r* = -0.231, *p* < .001), reflecting that males were on average older (mean = 37.10 years, SD = 11.46) than females (mean age = 31.93 years, SD = 1.19) in our sample.

**Table 3:** Correlations between key model predictors.

	Age		Gender (female 1, male 2)		Risk propensity (RPS)		Handedness (left/ambi 1 right 2)		Optimism bias	
	*r*	*p*	*r*	*p*	*r*	*p*	*r*	*p*	*r*	*p*
Numerical ability*	-0.048	.423	-0.002	0.968	0.074	.212	0.014	.818	-0.075	.212
Age			-0.231	< .001	-0.112	.059	0.091	.126	0.137	.021
Gender					0.275	< .001	-0.012	.843	-0.012	.841
Risk propensity							-0.205	< .001	0.037	.518
Handedness									-0.010	.865

*Average error on Number Line task.

### MicroMort acute risk perception task

**Fig 1:**
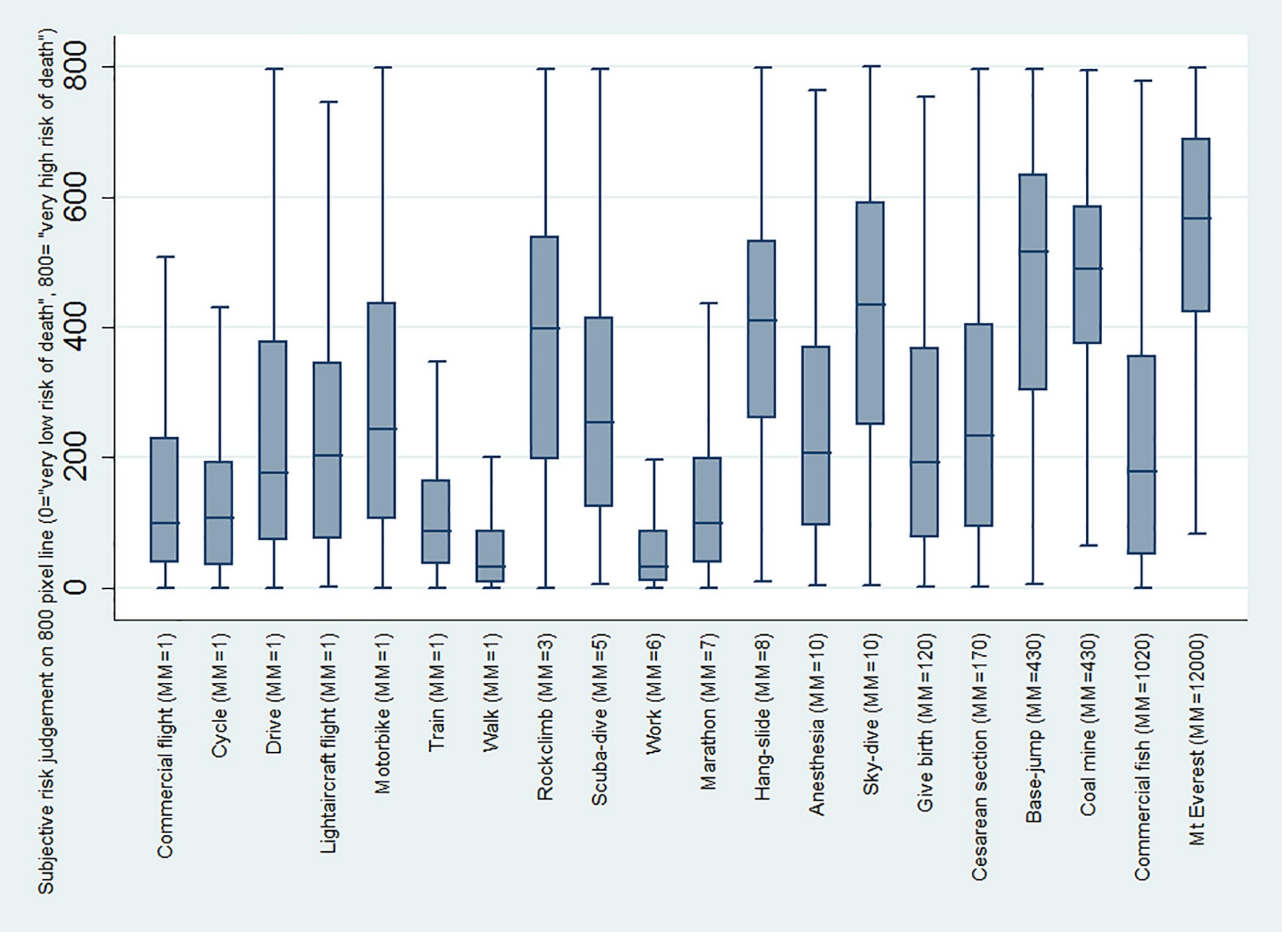
Box-plot of subjective risk perception on the 800-pixel line (i.e. responses varied between 0 and 800) for all 20 activities. Each box represents the interquartile range of subjective risk perception judgements for each activity, with the median crossing within each box, and all data points within 1.5 of the upper and lower interquartile ranges are represented by the whiskers. MM = MicroMort value.

Fig 1 displays a box-plot of subjective risk perception judgments for all 20 activities. Despite subjective judgments generally increasing as objective MicroMorts increase, it is obvious that there is wide variation (even within the activities with a MicroMort value of 1).

**Fig 2:**
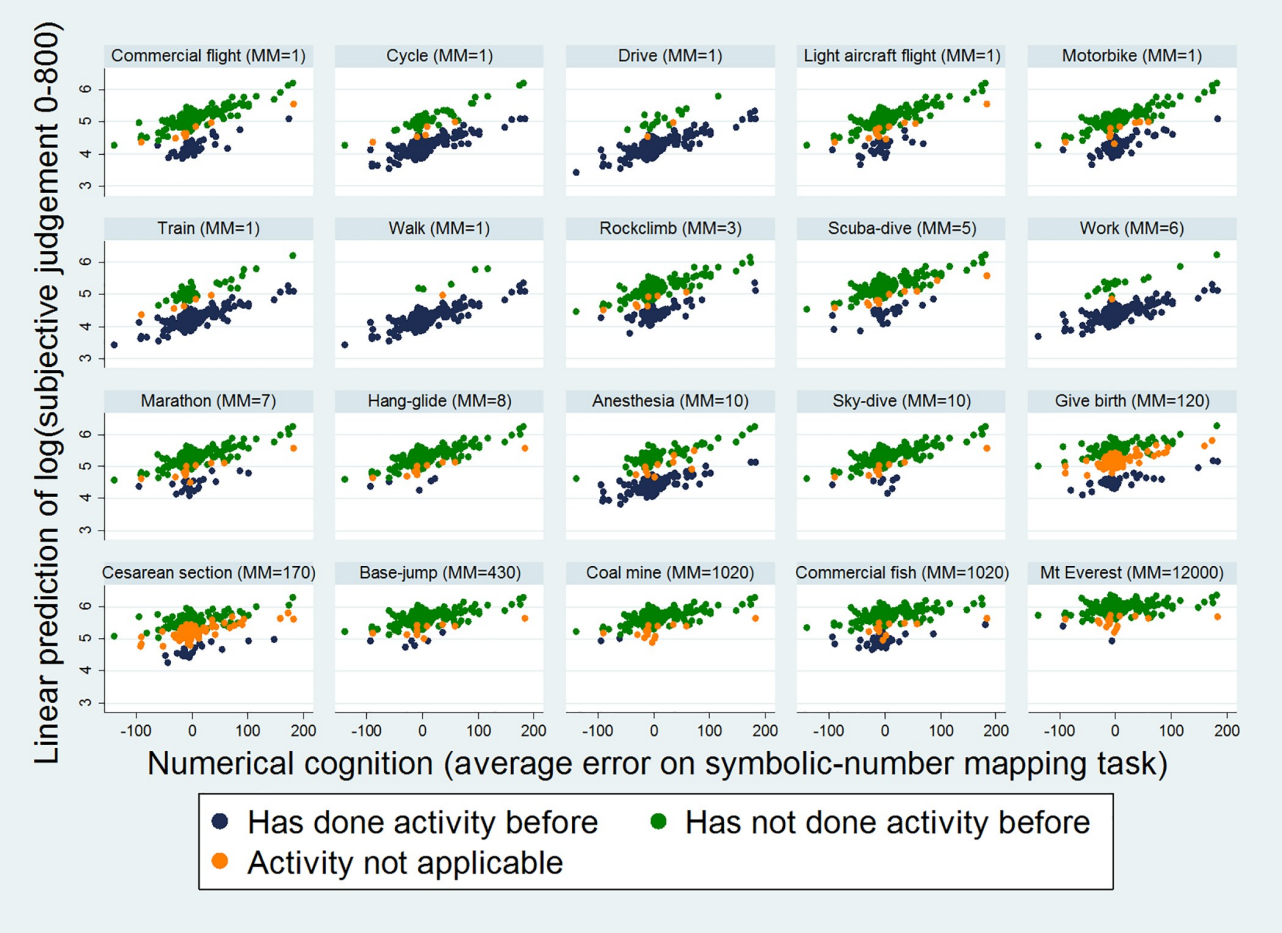
Scatter plots of linear predicted values (from fixed portion of mixed effects model; of log of 1 + judgment on 800 pixel line) relative to activities varying in their objective MicroMort (MM) risk, numerical ability (average error on symbolic-number mapping task) and prior experience. Higher values on the y axis represent a higher perceived risk. It can be seen that those who underestimate on the symbolic-number mapping task, perceive risks to be lower, especially for low risk (smaller MicroMorts) activities; and further, those who have undertaken the task previously, perceive risks to be smaller.

From Table 4, it can be seen that significant predictors of subjective risk perception (i.e. performance on the MicroMort task) in the mixed effects model were objective MicroMort value, indicating that individuals did estimate risk to be greater for activities that were objectively riskier; numerical ability as indexed by average error on the symbolic-line task was also positively associated, with those who under-estimated provided lower subjective risk perception judgments than those who over-estimated; the interaction between objective MicroMorts and numerical ability, with this effect, indicating that numerical ability associated with subjective risk perception less for objectively high-risk activities, as compared to objectively low-risk activities; and lastly, prior experience with the activity was associated with lower subjective judgments than no experience, or deeming the activity to be not applicable. See Fig 2 for a graphical illustration of significant effects. Gender, handedness, optimism bias, risk propensity did not significantly associate with subjective risk perception in the full model. The effect for age missed conventional significance levels (*p* = .062), with older adults judging risks to be lower, although not statistically significantly.

**Table 4:** Fixed effects for model predicting subjective risk perception (log of 1 + response on 800-pixel length line).

Predictor	Coefficient	z	*p*	95%CI	*f*^2^
Intercept	4.5383	13.06	< .001	3.8572–5.2194	
Objective MicroMort	0.0954	16.78	< .001	0.0842–0.1065	.0530
Numerical ability (average error on task)	0.0051	4.86	< .001	0.0030–0.0072	.0005
Objective MicroMort * numerical ability	-0.0004	-3.38	.001	-0.0007 –-0.0002	.0022
Previous experience					.1216
Yes (referent)	–	–	–	–	
No	0.8680	25.12	< .001	0.8003–0.9357	
Not applicable	0.4598	6.22	< .001	0.3148–0.6048	
Age	-0.0070	-1.86	.062	-0.0144–0.0004	< .0001
Gender (female 1, male 2)	0.1047	1.21	.226	-0.0646–0.2739	< .0001
Risk propensity	0.0047	1.13	.258	-0.0034–0.0127	< .0001
Handedness (left/ambi. 1, right 2)	-0.1918	-1.36	.173	-0.4679–0.0843	< .0001
Optimism bias	-0.0028	-1.07	.283	-0.0078–0.0023	< .0001

## Discussion

Participants did judge objectively riskier everyday activities as subjectively more risky, although the effect size was small and they were very poor at judging the relative differences of the acute risk of death conveyed by each activity. For example, as compared to walking 27 miles (MicroMort of 1), participants judged going under general anaesthesia (on average) only four times more risky, yet it carries a ten fold risk; and climbing Mt Everest as over nine times as risky yet carries a 12,000 fold risk. This finding reflects broader decision making theories, where we overweight probabilities of small risks and underweight large risks probabilities [8, 9].

This pattern of misjudging risks bears resemblance to the judgments of numerical distances. Distances between large numbers (e.g., distance between 1,001 and 1,005) are underestimated in comparison to distance judgments between small numbers (e.g., distance between 1 and 5). In other words, the subjectively perceived distance between numbers varies as a function of the numbers’ magnitudes: equal distances between larger magnitudes are perceived to be smaller than the same distances between smaller numbers [54]. One common explanation for this size effect is that the mental representation of magnitudes is not linear, with the representation of larger numbers being compressed [54]. The current findings of underestimated high-risk activities (e.g., climbing Mt Everest) suggests that the mental representations of everyday risk and number magnitudes share similar characteristics.

In a similar vein, performance on the numerical judgment task was independently related to subjective judgment of everyday activities, with a small effect size. Individuals who numerically under-estimated provided lower subjective risk perception judgments than those who over-estimated. While speculative, the observed relationship between number and everyday risk estimates are in line with the idea of a common magnitude representation system in the brain [41]. Given the preliminary nature of these data however, the relationships risk perception and numerical abilities, including the mental number line, should be an avenue of future research.

The sense of numerical magnitudes is widely thought to depend on a non-verbal quantity representation system located in the parietal lobes of the brain [55]. If asked to identify the larger of two numbers (e.g., 4 and 9) or to judge distances between two numbers we rely on this non-verbal approximate number system (ANS). There are suggestions that this quantity system might be part of a general magnitude system which also mediates the judgment of non-numerical magnitudes [41, 42]. That is, judgments on the size, distance and length of properties such as time, space, luminance, and numbers might rely on the same magnitude system in the brain [41–43]. Further studies are required to seek confirmatory evidence that there is indeed such a common magnitude representation system and that risk magnitudes are mapped onto this system.

The aforementioned view relies on the popular account that our understanding of numbers and magnitudes relies on the ANS. It is important to acknowledge, however, that there are alternative accounts which question that numerical symbols map onto the ANS [56]. Moreover, this exploratory study only employed the symbolic-number mapping (number line) task. The number line task measures some aspects of numerical competence and has been related to measures of numeracy (e.g., [45, 57]), but it is not a pure measure of magnitude estimation and task performance is influenced by proportional judgments [58, 59]. Hence, while the current results suggest that some aspects of numerical competence are related to the accuracy of everyday risk judgments, any links to the ANS and the generalized magnitude systems must remain tentative at this stage.

Notably, the association between numerical ability and subjective risk perception attenuated as objective risk increased, indexed by an interaction (between performance on the Number Line task and objective MicroMort value) with a small effect size. That is, numerical ability was associated with risk perception mostly strongly for low risk stimuli.

Previous experience with the activity was found to associate with everyday risk perception judgments, with a small-medium effect size (the largest of all model predictors). Having undertaken the activity before was associated with a reduction in risk perception as compared to not having done the activity, or deeming it not applicable. This corresponds with previous studies reporting that smokers underestimate the mortality effects of smoking [27] along with lung cancer, heart attack and stroke [2, 3].

Agans and Shaffer [60] reported that in an experimental study using risk-related stories, participants were capable of making relatively appropriate probability estimates for disease, accident and homicide in foresight (i.e. with no outcome information), but they made relatively biased estimates in hindsight (i.e. with outcome information provided). They suggested that hindsight/outcome information invokes the use of the availability heuristic, whereby people rely on the knowledge of the outcome rather than statistical probabilities. Applying the availability heuristic to our findings could mean that those who have completed the activity without death as an outcome rely on this experience, rather than more objectively accurate known probabilities. Related, Renn [61] stated that one intuitive bias of risk perception was avoidance of cognitive dissonance, where information that questions perceived probabilities that are part of their belief system will be ignored or attenuated.

Another finding could also explain the effect of previous experience on risk perception in the literature: individuals judge themselves to perform better on average for easy tasks and worse than average for hard tasks [62, 63]. Moore and Cain [62] proposed that this was due to individuals having better information about themselves than they do about others, and therefore for harder (and less experienced) tasks, their judgments of others performance is less extreme (more regressive to the mean) than for easier (and more experienced) tasks.

Previous experience, as measured in this study, assessed personal experiences with the activity. Knowledge of others’ previous experiences [38], and expertise with the topic [64], may also affect risk perception. Rutter et al. [38] reported that a history of motorbike accidents did not predict personal perceptions of motorcycling risks (injury or death) in a large sample of motorcyclists, however, having a family member or friend who had been seriously injured or killed during motorcycling was positively related. Friedmann et al. [64] demonstrated that general physicians perceived the risk of their patients’ cardiovascular risk (without treatment) to be higher, and the benefit of pharmacological treatment to be greater, than cardiologists (specialists); judgments made by cardiologists were more accurate. An avenue of future research would be to assess the interactive effects of personal experiences, experiences of others’, and expertise with the risk, on risk perception.

Additional factors investigated in this study (age, risk propensity, and unrealistic optimism) were not related to the everyday risk estimates. These factors have previously been linked to gambling- and health-related risk judgments [2, 28, 65, 66]. For example, Dillard et al. [66] reported that smokers who were unrealistically optimistic (regarding risk of lung cancer) were more likely to endorse false risk-related statements (e.g. no risk of lung cancer if one only smokes for a few years, and that getting lung cancer depends on one's genes); they were also less likely to plan on quitting smoking. The lack of significant association between risk propensity and performance on the MicroMort task was surprising, however, may reflect the domain-specificity of risk-taking and judgment. The risk propensity scale is generic regarding the risk domain (e.g. recreational, social, financial, health), while our MicroMort task related to recreational risks related to everyday activities. Risk-taking has been shown to be domains specific, and using a scale such as the Domain-Specific Risk-taking scale (DOSPERT) may be useful in future research [28, 67]. While the current results need to be confirmed, they support the notion that individual differences underlying risk perception vary across risk domains such as gambling/financial, health and recreation.

This study is not without limitations. MicroMorts are averages and do not reflect individual circumstances. However this study employed a large sample size, meaning that these individual circumstances carry less weight. It must also be acknowledged that the MicroMort acute risk perception task was procedurally identical to the number line task: they both involved clicking points on a horizontal line. It may be possible that an unmeasured individual difference unrelated to magnitude estimation was driving responses on these line tasks [58]. Future research should use a broader array of tasks with different response requirements as our experimental design cannot rule out the possibility that non-numerical processes related to responding on a horizontal line might explain the relationship between the tasks.

Also in relation to the design of the MicroMort and number line tasks, participants could only overestimate but not underestimate small numbers. These response patterns produce nonhomogeneous errors, which are better suited to Bayesian approaches. We hope our findings can be used to determine priors for future Bayesian work in the area. Lastly in relation to the line task, an 800 pixel line was selected as it could be displayed on the vast majority of screens, however, it imposed errors in terms of subjective responses and statistical estimates of both numbers and risk. As for both numbers and risk activities, the objective scale was larger than 800.

We did also not take chronic risk into account, such as that conveyed by obesity or heart disease, which affect lifespan rather than the acute risk of death. Also, individual traits and behaviours (e.g. tendency for harm avoidance in Obsessive Compulsive Disorder [68]) were not assessed. And finally, commercial fishing may have been misunderstood as fishing in general, looking at response frequencies, which likely contributed to the underestimation of risk. A positive of our approach is the use of the MicroMort framework that enables us to index acute risk of death for everyday activities. Previous research has focused on health [2, 3] or gambling-related risk perception [69], and identifying associated individual differences such as numerical ability [26] in these contexts. Micromorts are a unique framework, providing objective risks for everyday activities, and could be utilised more often in psychological research.

Risk is inherent in every judgment we make and people need to understand risk to make informed choices. To improve judgments and choices, we need to effectively communicate risks, which is especially important in medical consent [32]. To do so, the current findings suggest that the observed link between numerical and risk magnitude processing should be further investigated. From a theoretical point of view it is important to further investigate whether risk processing taps into a common magnitude processing system. It would be interesting to know whether risk magnitudes are implicitly mapped on space in a similar manner as numbers and other quantities are. Lastly, it is important to establish whether risk perception (including that related to Micromorts) are amendable to training/feedback [70]. Positive answers to these theoretical points could inform everyday risk communication [34]. Visualisation and graphical strategies are known effective communication tools [71]. In a randomised control trial, Fagerlin et al. [72] showed that designed risk communication led to participants being less influenced by misleading anecdotes, which is an important finding. This raises the question whether integrating the current knowledge about the processing of magnitudes to visual and graphical communication strategies could lead to even better risk communications.

This study has shown that the MicroMort framework can be used to assess subjective risk perception judgments, which enables researchers to investigate risk perception in relation to recreation, as opposed to finance and health. This is an important step. Findings illustrate that we can judge what activities are riskier than others, however, we are poor at judging the relative risks differences of activities. Undertaking the activity previously was related with lower risk perception. The association between the misjudging of both risks and numerical magnitudes warrants further investigation with a broad range of numerical tests, as the finding could have large implications for theories of magnitude representation in the brain and risk communication.

## Supporting information

S1 Data Data underlying this study. (XLSX)
